# Roadside verges and cemeteries: Comparative analysis of anthropogenic orchid habitats in the Eastern Mediterranean

**DOI:** 10.1002/ece3.5245

**Published:** 2019-05-22

**Authors:** Réka Fekete, Viktor Löki, Renáta Urgyán, Kristóf Süveges, Ádám Lovas‐Kiss, Orsolya Vincze, Attila Molnár V.

**Affiliations:** ^1^ Department of Botany University of Debrecen Debrecen Hungary; ^2^ Department of Tisza River Research MTA Centre for Ecological Research‐DRI Debrecen Hungary; ^3^ Evolutionary Ecology Group, Hungarian Department of Biology and Ecology Babeş‐Bolyai University Cluj‐Napoca Romania

**Keywords:** burial spaces, Cyprus, Greece, Orchidaceae, roadsides, secondary habitats

## Abstract

Several important habitats have become threatened in the last few centuries in the Mediterranean Basin due to major changes adopted in land‐use practices. The consequent loss of natural and seminatural orchid habitats leads to the appreciation of small anthropogenic habitats, such as cemeteries and roadside verges. Colonization of cemeteries and roadside verges by orchids has long been known, but no study to date compared the suitability of these two anthropogenic habitats for orchids. Therefore, in this paper our aim was to survey cemeteries and roadside verges and to compare these two habitats regarding their role in conserving Mediterranean terrestrial orchids. We conducted field surveys in three Mediterranean islands, Cyprus, Crete, and Lesbos, where both cemeteries and roadside verges were sampled on a geographically representative scale. We found a total of almost 7,000 orchid individuals, belonging to 77 species in the two anthropogenic habitat types. Roadside verges hosted significantly more individuals than cemeteries in Crete and Lesbos, and significantly more species across all three islands. Our results suggest that although cemeteries have a great potential conservation value in other parts of the world, intensive maintenance practices that characterized cemeteries in these three islands renders them unable to sustain valuable plant communities. On the other hand, roadside verges play a prominent role in the conservation of Mediterranean orchids in Cyprus and Greece. The pioneer status of roadside verges facilitates their fast colonization, while roads serve as ecological corridors in fragmented landscapes.

## 1 INTRODUCTION

Mediterranean landscapes are the result of complex interactions between society and ecosystems throughout the millennia (Thompson, 2005). Some of these landscapes are of paramount importance. For instance, the grasslands of the Mediterranean are among the world's biodiversity hotspots (Sirami et al., 2010). Traditional land‐use practices characterized by frequent and moderate disturbances such as wood‐cutting and coppicing, terracing, controlled burning, grazing, and browsing helped to preserve these Mediterranean habitats for a long time (Blondel, 2006). Nonetheless, this ecological equilibria and diversity of endemic species maintained by the traditional agro‐sylvo‐pastoral land use has been disturbed recently. During the last few decades, traditional land‐use practices have been abandoned in many places, leading to an overall decrease in biodiversity (Bignal & McCracken, 1996; Blondel & Aronson, 1999; Lasanta‐Martínez, Vicente‐Serrano, & Cuadrat‐Prats, 2005). Besides abandonment of traditional land‐use practices, major changes occurred in land use due to urbanization, agricultural intensification, and intensifying tourism (Krauss et al., 2010; Medail & Quezel, 1997; Myers, Mittermeier, Mittermeier, da Fonseca, & Kent, 2000; Nascimbene, Zottini, Ivan, Casagrande, & Marini, 2016; Tikka, Koski, Kivelä, & Kuitunen, 2000; Tilman et al., 2001). For instance, the remarkable orchid habitats of the Mediterranean Islands, such as phrygana, maquis, olive grows, vineyard terraces, salty meadows, coastal wetlands, and pine forest have recently become threatened biotopes. The causes include overgrazing or lack of grazing, intensification of cultivation (especially in the case of olive groves), growing tourism at coastal areas, agricultural use of rivers for watering (leading to local drought) (Kretzschmar, Kretzschmar, & Eccarius, 2004; Kreutz, 2004). Therefore, small patches of remnant, seminatural vegetation with multiple threatened species became of conservation concern. These can sustain threatened species in anthropogenically influenced habitats, such as midfield islets and roadside verges (Cousins, 2006; Godefroid, 1999), cemeteries (Barrett & Barrett, 2001), freshwater pools and lakes (Lukács, Sramkó, & Molnár, 2013; Lukács et al., 2015), river dikes (Bátori et al., 2016), and kurgans (Deák, Tóthmérész, et al., 2016; Deák, Valkó, Török, & Tóthmérész, 2016).

Cemeteries have significant conservation importance (Buchholz et al., 2016; Czarna & Nowinska, 2010; Özhatay & Gürdal, 2013; Yılmaz, Kuşak, & Akkemik, 2018), especially in landscapes fragmented by urbanization (Barrett & Barrett, 2001). Cemeteries can act as refuges for natural vegetation due to cultural taboos against the disturbance of burial places (Hadi, Ibrar, & Zaidi, 2014). Furthermore, cemeteries often lack grazing and trampling pressure by animals, usually being fenced off from the surrounding area. During the past few decades, several orchid taxa were documented from cemeteries (Kreutz & Krüger, 2014; Löki et al., 2015), while some taxa have been found for the first time to the region in these anthropogenic habitats (Kreutz, 2010, 2013; Kreutz & Peter, 2007).

Roadside verges can also reserve valuable communities of the native flora and often serve as refugia in many places throughout Europe (Auestad, Rydgren, & Austad, 2011; Coffin, 2007; Deckers, Becker, Honnay, Hermy, & Muys, 2005; Fekete et al., 2017; Hovd & Skogen, 2005; Hussey, 1999; Vasconcelos, Araújo, & Bruna, 2014). Moreover, the linear structure of roads can act as dispersal corridors for plants (Tikka, Högmander, & Koski, 2001), being especially relevant to small, light seeded species that can disperse by wind turbulence, or within the soil adhered to vehicles (Clifford, 1959; Ross, 1986). Moreover, constructions and road cuttings often create free soil surfaces, which are suitable places for pioneer species such as orchids (Arditti & Ghani, 2000; Murren & Ellison, 1998). Colonization of roadside verges by orchids is a phenomenon that has long been known (Federici & Serpieri, 1868; Good, 1936; Turrill, 1932). Surveys of roadside verges of two Mediterranean islands (Corfu and Mallorca) revealed the presence of 12 orchid taxa in these habitat patches (Brandes, 1998a, 1998b).

The aims of this paper were to survey and compare two kinds of anthropogenic orchid habitats (cemeteries and roadside verges) in three Mediterranean islands: Cyprus, Crete, and Lesbos. We aimed to (a) study whether cemeteries or roadside verges host more orchid individuals and species, and which one of these play a more significant role in orchid conservation; (b) test, which environmental factors influence the prevalence of orchids in these two synanthropic habitats; (c) examine, if there is an anthropogenic effect on the abundance of orchids, reflected by the proximity to human settlements; and (d) test whether the proximity of road has a negative effect on the occurrence of orchid individuals.

## 2 MATERIALS AND METHODS

### 2.1 Field work

Field samplings were carried out in three Mediterranean islands, Cyprus (area of Republic of Cyprus), Crete (Greece), and Lesbos (Greece, Table 1). We surveyed cemeteries and roadside verges in all three islands. Surveyed cemeteries were randomly selected, but in a geographically representative manner. For each visited cemetery, we recorded geocoordinates (WGS84 format) and altitude (m) using Garmin E‐Trex Legend GPS device. Then, we carried out a thorough search for orchids covering the entire area of the cemetery, identified the species of each orchid found and recorded the number of specimens belonging to each of these (following Löki et al., 2015). We additionally measured the total area of the cemetery, and the area covered by graves, concrete (e.g., paved areas, paved paths), forest, and grassland using the Google Earth Pro software (Google Earth, 2018). Concrete, forest, grassland cover, as well as area occupied by graves were expressed relative to the overall area of the cemetery (i.e., 0%–100%).

**Table 1:** Summary of sampled roadside verges and cemeteries, sampling methods and dates of the field surveys

Site	Sampling date	Number of non‐thematically sampled roadside verges	Number of thematically sampled roadside verges	Number of sampled cemeteries
Cyprus	11–22 March, 2016	56	0	57
Cyprus	16–19 May, 2016	1	0	1
Cyprus	29 March–04 April, 2017	6	120	32
Crete	11–19 April, 2017	58	120	90
Lesbos	9–17 April, 2018	14	204	35

Two types of sampling processes were adopted in the case of roadside verges. First, we conducted thematic sampling by driving along asphalt roads and we stopped every 5 km in Cyprus and Crete. A shorter section length (i.e., 2 km) was defined in the case of Lesbos, due to the small area of the island compared to the other two sampled islands. Second, we conducted nonthematic sampling, meaning that we stopped at every road section, where orchids were spotted from the car. At every sampling point, we recorded geocoordinates (WGS84 format) and altitude (m) using the above mentioned GPS device. In the case of thematic and nonthematic sampling points, we measured different environmental factors, which could influence orchid presence, such as the angle of slope, tree, and shrub cover, and width of the roadside verge, which differed among the sites. Width of the roadside verges was measured for calculating the total area of the sampling point at roadside verges. In case where orchids were present these parameters were recorded for every individual, while if orchids were absent the parameters were recorded every 10 m along a 50 m road section. Where orchids were found, we additionally recorded the detected orchid species and the number of specimens belonging to each of these along a 50 m road section and the distance of each individual from the edge of the road. Measurements of angle of slope, tree, and shrub cover, as well as width of the roadside verge were averaged per sampling site prior to the statistical analyses. In some cases, identification of orchids to the species level was not possible, due to their vegetative physiological state. In such cases, the number of these individuals was counted and was used in the analyses regarding the number of orchid individuals, but was ignored in the analyses regarding the number of orchid species (see below). A total of 381 individuals, found at 41 locations could not be identified to the species level.

Furthermore, for all three types of sampling points we measured the distances between the point and the closest human settlement (the closest building of the nearest settlement to the sampling point) in straight line and on road using Google Maps in the case of all three islands.

Taxa were identified following Kreutz (2004), Kretzschmar et al. (2004) and Delforge (2006). Total number of orchid species occurring in Cyprus and Crete were quantified based on Delforge (2006), while in Lesbos based on Karatzas and Karatza (2009). In this paper, we follow the nomenclature of Delforge (2006).

### 2.2 Data analyses

Statistical analyses were carried out in R statistical environment (version 3.4.1, R Core Team, 2017). For the analyses, we built generalized linear mixed models (GLMM) with negative binomial error distribution, using the *glmmTMB* function (*glmmTMB* R package, Brooks et al., 2017). Zero‐inflated models were applied in cases where including the zero‐inflation parameter increased model fit, as indicated by lower Akaike information criterion (Wagenmakers & Farrell, 2004). In all cases, we started by building full models containing all explanatory variables. This was followed by model simplification, when nonsignificant predictors were removed from the model using a stepwise backward procedure, based on the largest p values, until minimally adequate models were obtained.

First, we tested whether cemeteries or roadside verges host more orchids using GLMMs. For these models, only thematic sampling points of roadsides and cemeteries were used. In the case of cemeteries, all 90 sampling points were included from Crete, all 35 from Lesbos, but in the case of Cyprus, we used 89 cemeteries in the analyses, and excluded one, due to the latter being sampled a month later than all the others. The number of species and the number of individuals detected at each sampling point were used as dependent variables in subsequent models. Site (cemetery or roadside verge) and the area of the sampling point were included as explanatory variables, while island was included as a random factor in the models.

To test how environmental factors influence the colonization success of orchids in cemeteries, we built GLMMs. In these models, the number of orchid species and the number of orchid individuals were used as dependent variables, while explanatory variables included total area of the cemetery, the proportion of total area covered by concrete, forest, grassland, as well as the proportion of area occupied by graves. We built similar models for roadside verges, but due to the excess of zeros zero‐inflated GLMMs were used here. The number of individuals and the number of species detected at each sampling point were used as dependent variables in subsequent models. In these models, data from both thematic and nonthematic sampling points were included. Mean angle of slope, area of the sampling points, as well as mean tree and shrub cover were included as explanatory variables, while island and sampling type were included as random factors in the models.

Furthermore, to test how the distance to the closest settlement influences the number of orchid individuals and on the number of orchid species, we built GLMM models with negative binomial error distribution, using the *glmmTMB* function. We used the number of species and the number of individuals detected at the sampling points as dependent variables in consecutive models. Explanatory variables were the area of cemeteries or roadside verges, the distance to the closest settlement in straight line and on road, the interaction between site (roadside or cemetery) and distance in straight line and the interaction between site and distance on road. Island and sampling type (i.e., thematic/nonthematic) were included in the models as random factors. In the latter analyses, we used data derived from both thematic and nonthematic samplings.

In order to test the null hypothesis that absolute and relative distance from road follows a uniform distribution we used One‐sample Kolmogorov–Smirnov tests. The latter test was performed for the three islands separately.

## 3 RESULTS

### 3.1 Basic summary of orchids of the three islands from the two different habitats

During the surveys in Cyprus, Crete, and Lesbos, we found a total of 6,962 orchid individuals; 1,424 in cemeteries and 5,538 on roadside verges. These orchids belonged to 77 different species (Table S1). The highest number of orchid individuals was found in Crete (3,373), followed by Cyprus (2,454) and the lowest number of individuals was found in Lesbos (1,135). Similarly, the species diversity of the detected orchids was the richest in Crete (41), followed by Cyprus (32), and the lowest number of species was detected in Lesbos (31). In the case of three genera (*Anacamptis* sp., *Ophrys* sp., *Serapias* sp.), identification to the species level was not possible at 41 sampling points.

Overall, the most abundant orchid species were *Serapias bergonii*, *Orchis sancta,* and *Orchis fragrans*. The orchid represented with the highest number of individuals was the *Himantoglossum robertianum* (331) in Cyprus, *O. fragrans* (676) in Crete, and *O. sancta* (608) in Lesbos.

### 3.2 Cyprus

**Figure 1:**
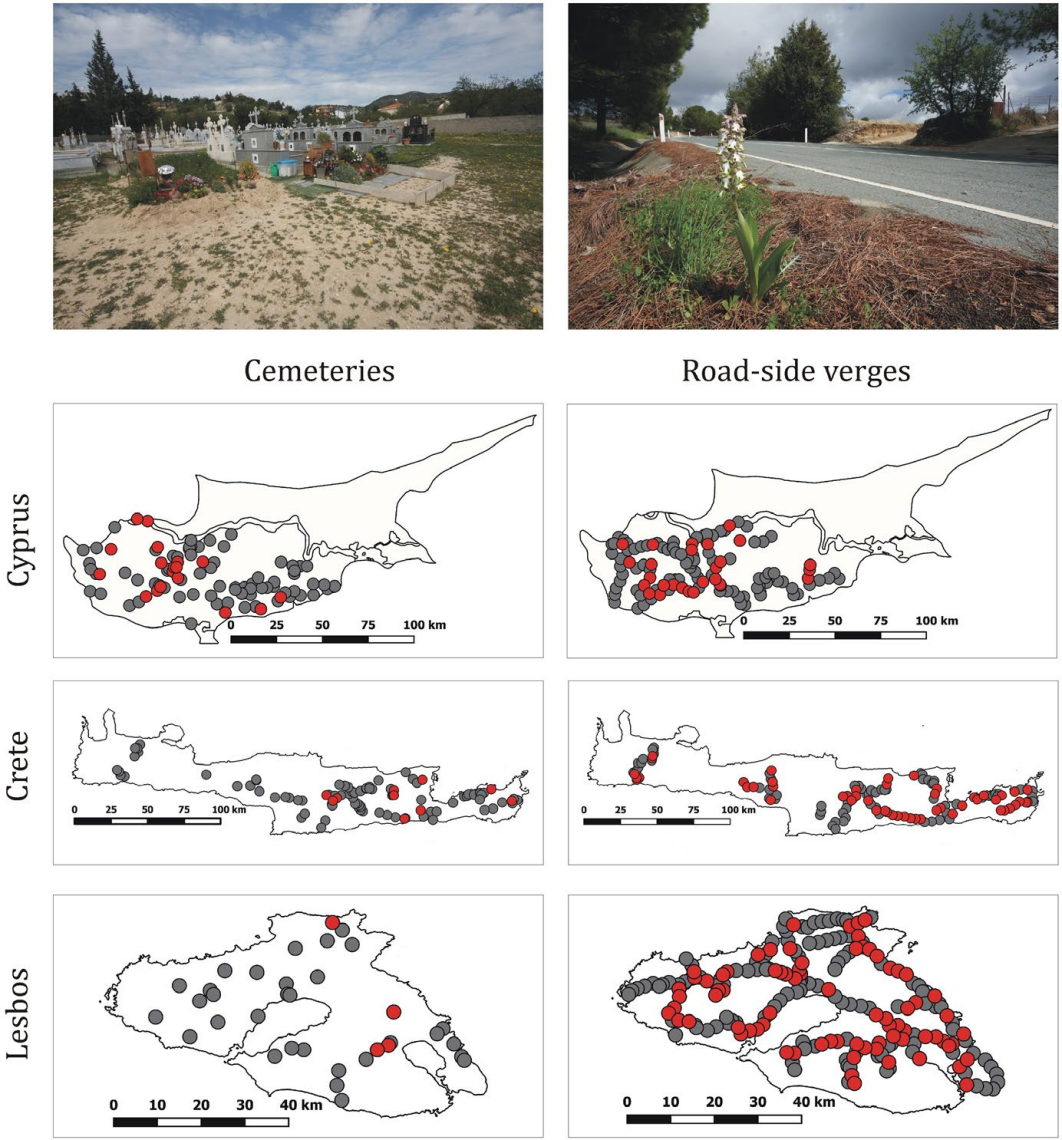
Geographic location of visited cemeteries (left column) and thematic sampling points on roadside verges (right column) in Cyprus, Crete, and Lesbos, respectively. Gray dots indicate the absence, while red dots the presence of orchids

In Cyprus, we found orchids in 20% of the cemeteries (Figure 1, Table 2). The most abundant species in Cyprus was the *H. robertianum* that was present at the 7% of the visited cemeteries. Species with the highest number of individuals present in cemeteries were the *Ophrys flavomarginata* (150 individuals) and the *Orchis collina* (102 individuals).

**Table 2:** Comparison of thematic sampling of cemeteries and roadside verges of the three islands regarding their importance as orchid habitats

	Cyprus		Crete		Lesbos	
	Cemetery	Roadsides	Cemetery	Roadsides	Cemetery	Roadsides
Number of sampling points	89	120	90	120	35	204
Ratio of sampling points with orchids	20%	23%	12%	42%	11%	37%
Mean number of species	0.11	0.16	0.14	0.28	0.09	0.11
Mean number of individuals	6.02	4.84	9.41	11.18	1	3.98
Total number of species	10	20	13	34	3	23
Total number of individuals	542	581	847	1,342	35	812
Representation of the total orchid flora of the island	19%	38%	19%	49%	5%	39%

In respect to roadside verges of Cyprus, we found orchids at 27 thematic sampling points (Figure 1, Table 2). During nonthematic sampling, 1,034 individuals of 31 species were detected at roadside verges. Most abundant species of Cyprian roadside verges was *H. robertianum* (307 individuals), which was present in the 31% of the sampling points. The two other species with the most number of individuals were *S. bergonii* (276 individuals) and *Ophrys alasiatica* (230 individuals).

### 3.3 Crete

In Crete, orchids were found in 12% of the surveyed cemeteries (Figure 1). Most abundant species were *Ophrys sicula, Orchis talic*, and *Orchis papilionacea*, which were present in the 3% of the visited cemeteries. Species with the highest number of individuals were *O. fragrans* (500 individuals) and *S. bergonii* (276 individuals).

At roadside verges, orchids were found at 51 thematic sampling points (Figure 1). We found 1,184 individuals belonging to 39 species at 58 nonthematic sampling points of roadsides in Crete. Most abundant species of Cretan roadside verges were the *S. parviflora*, which was present in the 47% of the sampling points. Species with the highest number of individuals were *S. bergonii* (354 individuals), *O. collina* (254 individuals), and the *H. robertianum* (230 individuals). More characteristics of cemeteries and roadside verges of Crete are shown in Table 2.

### 3.4 Lesbos

On the island of Lesbos, we found orchids in 11% of the visited cemeteries (Figure 1). *O. sancta* was the most abundant species of cemeteries of the island, represented by 30 individuals and was found in the 8% of the visited cemeteries. During thematic sampling, we found orchids at 76 locations at roadside verges (Figure 1, Table 2). We detected 279 individuals belonging to 22 species at 14 nonthematic sampling locations. Most abundant species of roadside verges in Lesbos were *O. sancta* (578 individuals), which was present in 28% of the sampling points. Two other species with the highest number of individuals were *Serapias parviflora* (184 individuals) and *Ophrys sicula* (148 individuals).

### 3.5 General statistics including all three islands

Results of the GLMM explaining variation in the number of orchid individuals and the number of orchid species indicated that with increasing area of the sampling points both the number of specimens and the number of individuals increases significantly (Table 3). Moreover, there is a significant difference between types of sampling sites both regarding the number of orchid individuals and the number of orchid species. The latter result indicates that roadside verges host a significantly more abundant and more diverse orchid community than cemeteries.

**Table 3:** Results of GLMM explaining variation in the number of orchid individuals and species in response to the area and type (cemetery/roadside) of the sampling points

	Estimate	*SE*	*z* Value	*p* Value
Number of individuals				
Intercept	2.36	0.40	5.91	<0.001
Area	0.68	0.16	4.25	<0.001
Site ‐Roadside	2.24	0.50	4.45	<0.001
Number of species				
Intercept	−0.93	0.32	−2.93	0.003
Area	0.31	0.12	2.54	0.011
Site ‐Roadside	1.93	0.36	5.43	<0.001

Regarding the role of cemetery characteristics in the colonization success of orchids, our results highlighted the importance of forest and grassland cover. Forest cover had a significant positive effect on the number of species, while grassland cover had a marginally insignificant positive effect on the number of orchid species present. Moreover, concrete cover had a significant negative effect on the number of orchid individuals found in cemeteries (Table 4).

**Table 4:** Results of GLMM showing significant effects (and trends) of the measured parameters of cemeteries on the number of individuals and species (only minimal models are shown)

	Estimate	*SE*	*z* Value	*p* Value
Number of individuals				
Intercept	0.94	0.35	2.72	0.007
Total area	0.57	0.22	2.64	0.008
Area covered with concrete	−0.81	0.39	−2.07	0.038
Number of species				
Intercept	−1.58	0.20	−8.04	<0.001
Total area	0.28	0.15	1.89	0.059
Area covered with forest	0.37	0.18	2.09	0.037
Area covered with grassland	0.39	0.20	1.95	0.051

In the case of roadside verges, the angle of slope had a significant negative effect on the number of orchid individuals and species (Table 5).

**Table 5:** Results of GLMM showing significant effects of the angle of slope on the number of individuals and species on roadsides (only minimal models are shown)

	Estimate	*SE*	*z* Value	*p* Value
Number of individuals				
Intercept	4.36	1.11	3.94	<0.001
Total area	0.50	0.23	2.12	0.034
Angle of slope	−0.33	0.12	−2.73	0.006
Number of species				
Intercept	4.36	1.11	3.94	<0.001
Total area	0.50	0.23	2.12	0.034
Angle of slope	−0.33	0.12	−2.73	0.006

We found that the distance of sampling points to human settlements on road and in straight line did not affect the number of orchid individuals (*t* = 0.234, −0.302, *p* = 0.815, 0.762, *df* = 777) or species (*t* = 0.256, 0.056, *p* = 0.798 0.955, *df* = 777) found locally.

**Figure 2:**
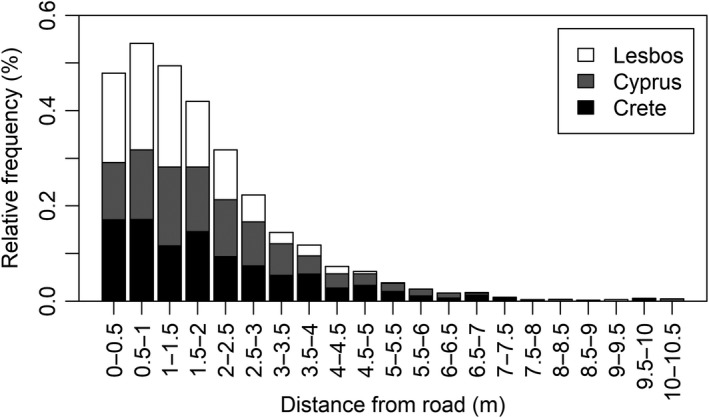
Distribution of relative distance from road of orchid individuals on all three islands

The spatial distribution of orchids along roadside edges did not follow a hypothetical uniform distribution in any of the three islands (Cyprus ‐ *D* = 733,380, Crete *D* = 6,581, *D* = 0. 0.5883, *p* < 0.001 in all three cases, Kolmogorov–Smirnov tests). Rather, individuals occurred closer to the edge of the road than expected by chance (Figure 2).

## 4 DISCUSSION

Our study highlights that orchids are present in large numbers and high diversity in both types of these little‐studied synanthropic habitats in three Mediterranean islands. During our surveys, altogether almost 7,000 orchid individuals were found belonging to 77 species, suggesting that cemeteries and roadside verges represent important habitats for several orchid species. However, cemeteries are considered as refugia for orchids in other Mediterranean regions, due to their intensive management in the surveyed islands our results indicate that roadside verges play a more important role in orchid conservation than cemeteries in Cyprus, Crete, and Lesbos, since we found fewer orchid specimens and a less diverse orchid flora in cemeteries than on roadside verges. The fact that cemeteries are also potential refugia for orchids shows the importance of more natural maintenance practices of these urban habitats.

Multiple possible hypotheses exist explaining the higher orchid diversity at roadsides, such as the intensive maintenance regimes in the surveyed cemeteries. Intensification of management may be attributed to cultural and religious reasons, given that previous studies have shown differences in orchid diversity between Christian and Muslim cemeteries in the Mediterranean (Molnár, Takács, et al., 2017). Moreover, a further study confirms the importance of Muslim cemeteries in the preservation of Mediterranean orchids (Löki et al., 2015). Therefore, religious differences between the islands surveyed here (mainly Christian) and countries surveyed by earlier studies might explain the lower orchid number and diversity reported here in comparison of previous reports (Molnár, Nagy, et al., 2017). It is apparent that religious differences determine several biotic and abiotic conditions of cemeteries, since according to Molnár, Takács, et al. (2017) Muslim cemeteries were significantly larger, than Christian ones, contained larger grassland areas and had a smaller proportion of area covered by graves. These characters might prevail due to the fact that certain anthropogenic activities, such as grazing mowing and cutting of plants are strictly prohibited in Muslim cemeteries. As a result, Muslim burial places represent more natural and inherently protected sites (Hadi et al., 2014), thus playing an important role in the maintenance of the native flora (Hadi, Akhtar, Shah, & Hussain, 2009; Rahman et al., 2007). The management of these burial places might often involve usage of herbicides, building of expensive large memorials and covering graves with large marble pebbles, marble, or concrete, excluding virtually all living organisms. The intensification of maintenance practices in cemeteries cannot be solely attributed to religious practices, social modernization and the adoption of Western cultures also have strong effects (Plumwood, 2007). Several studies highlighted that intensification of management practices leads to unfavorable processes that ultimately contribute to decreased biodiversity, and lowered conservation importance of cemeteries. Intensifying management involves the use of herbicides, frequent mowing or paving, soil disturbances (e.g., uprooting of trees) or planting ornamental plants, facilitating the colonization by non‐native species (Kowarik, Buchholz, Lippe, & Seitz, 2016; Stowe, Schmidt, & Green, 2001). Cemeteries on the other hand represent habitats where remnants of the ancient native vegetation supposedly been present for centuries and they have been conserved here due to the relatively constant environment over the years, in cases where management practices were appropriate (Molnár, Löki, et al., 2017). Our results also confirm the above mentioned impacts of intensification, since among the investigated characteristics of individual cemeteries, the relative area covered by forest, concrete, and grasslands were the best predictors of orchid densities and species richness in cemeteries. Increasing forest and grassland cover and decreasing concrete cover was associated with higher diversity and density of orchids in cemeteries. When the area of seminatural, green habitat patches like that of forests or grasslands decreases and paved, concrete areas increase, suitable habitats for orchids are disappearing, ultimately leading to lowered density and diversity of orchids. Consequently, preserving seminatural, green areas in cemeteries might play a key role in preserving the native flora.

Our results highlighted that roadside verges have significantly higher conservation potential than cemeteries, given that they appear to play a more important role in the conservation of several orchid species and individuals in the three surveyed Mediterranean islands. According to our surveys, Mediterranean roadside verges host numerous threatened orchid species. These include the least concerned *O. sancta* and *Epipactis veratrifolia*, the near threatened *Ophrys kotschyi*, and also the vulnerable *Orchis boryi* (IUCN, 2018). There are several reasons why orchids prefer roadside verges as habitats. For example, due to the weak competitive ability of most orchid species, they generally colonize newly created habitat patches (such as roadside verges), where the abundance of dominant plant species and the cover of trees and shrubs is low (Jersáková & Malinová, 2007). Furthermore, roadsides can act as ecotones, and due to the previously mentioned reasons, orchids frequently prefer these transitional areas (Bray & Wilson, 1992; Djordjević, Tsiftsis, Lakušić, Jovanović, & Stevanović, 2016; Duchoň, 2012; Rai, Adhikari, & Rawat, 2010; Slaviero, Del Vecchio, Pierce, Fantinato, & Buffa, 2016). The microscopic seeds of orchids are effectively dispersed by the wind, even on long distances (Arditti & Ghani, 2000), thus facilitates the effective colonization of new habitat patches, like newly created roadside verges, by these species. It seems that roadside verges are suitable linear habitats for several orchid species, but some of their characteristics could restrain their suitability to orchids. According to our survey, steep slopes had a significant negative effect on the number of orchid individuals and species present. This result might indicate the importance of hydrological properties of roadsides which might directly influence the suitability of the habitat for orchids. Steep slopes at roadside verges are exposed to the sun and are more prone to water runoff, therefore the soil here dries out easier and earlier (Bochet & García‐Fayos, 2004), creating microhabitats unsuitable for orchids. Beyond individual characteristics of cemeteries and roadside verges, other processes, such as urbanization could also have an impact on orchid diversity and density in these habitat patches.

Nowadays, urbanization is one of the biggest threats to biodiversity globally, causing habitat loss, fragmentation and ultimately a decrease in species diversity (Czech, Krausman, & Devers, 2000). Species are threatened by several physical alterations of their habitats, caused by human activities especially in urbanizing areas, including increasing human population and road density, air, and soil pollution, the “heat island” effect, soil compaction and alkalinity (McKinney, 2002). In contrast to these expectations, we did not find significant effects of the proximity of sampling points to human settlements, neither in the case of orchid individuals nor in the case of orchid species in the three surveyed Mediterranean Islands. In contrast, other anthropogenic activities, such as mowing of roadside verges could facilitate the survival of orchids at close proximity to road edges. This was confirmed by our results, what showed greater density of orchid individuals closer to road edge, and decreasing density with increasing distance from the road. Close proximity to roads might be beneficial for orchids because the immediate vicinity of roads is regularly mowed as part of road management. Due to mowing—which is known to have positive impact on orchids in other habitat types—the vegetation here is usually less closed, thus less competitive, and these conditions are favorable for species requiring high light intensities such as orchids for successful reproduction (Janečková, Wotavová, Schödelbauerová, Jarsaková, & Kindlmann, 2006; Sletvold, Øien, & Moen, 2010; Smith & Cross, 2016). However, close proximity to road could have a negative impact on reproductive success due to increased mortality of pollinators, road edges does not seem to be ecological traps to orchids (Fekete et al., 2017).

## 5 CONCLUSIONS

Our findings suggest that although cemeteries have a high conservation potential, intensive land management can lead to denaturalization in these habitats, eliminating remnants of the natural vegetation and rendering them incapable to preserve native species. Based on our results, preserving large green (forest and grassland) patches in cemeteries are favorable for orchid conservation, as these appear to shelter local orchids and provide adequate habitat patches for novel colonizations. In contrast, adopting paved walkways, as well as the usage of huge concrete graves represent disadvantageous management practices for the local flora and they lead to the decline of the natural vegetation over time. In contrast to modern burial trends, traditional burial mounds with small headstones and wooden crosses are more environmentally friendly. These modest graves give ground for the colonization of natural vegetation including orchids. Moreover, for conservation concerns, the use of herbicides should be avoided in cemeteries, as well as the planting of non‐native ornamentals, especially of species that have already spread rapidly in the region. On the contrary, planting native species on the graves and around them could be favorable. In contrast to cemeteries, roadside verges appear to currently function as refugia for several orchid species in this extremely fragmented landscape in the three surveyed islands. Moreover, these linear meadows act as dispersion corridors and they contribute considerably to landscape connectivity. Nonetheless, appropriate planning, building, and management of roads are very important to create and maintain roadside verges that are suitable for natural vegetation. Because of mild Mediterranean climate, de‐icing salt is rarely used in the surveyed islands, avoiding the negative impact of this chemical witnessed under colder climates, further increasing the conservation value of the studied roadside verges. We conclude that creating steep concrete retaining walls should be avoided and gentle slopes should be established instead, in order to form a gradual transition to the natural landform. Terracing with rock outcrops can support this by facilitating the establishment of vegetation and by creating microclimatic niches, while they stabilize the structure of road cuttings. Furthermore, our results indicate that mowing is the most favorable management practice of roadside verges from the orchid conservation point of view, since the regularly mowed 0–2 m margin of roads is the most important part of the roadside verge for orchid individuals. We strongly recommend avoiding paving road edges as well as the use of herbicides due to their unfavorable effect on the natural vegetation. Finally, we strongly urge local authorities to conduct appropriate field surveys and impact assessments prior to broadening roads.

## CONFLICT OF INTEREST

None declared.

## AUTHORS' CONTRIBUTIONS

AMV conceived the ideas and designed methodology; FR, LV, AMV, RU, KS, and ÁLK collected the data; FR and OV analyzed the data; FR, AMV, and OV led the writing of the manuscript. All authors contributed critically to the drafts and gave final approval for publication.

## Data Availability

All data used in the analyses are available from Dryad—http:/dx.doi:10.5061/dryad.f054b0d (Fekete et al., 2019).
