# Supplementary material for: Roadside verges and cemeteries: Comparative analysis of anthropogenic orchid habitats in the Eastern Mediterranean
Source: Ecol Evol. 2019 May 22;9(11):6655–64. doi: 10.1002/ece3.5245 (PMC6580262; doi:10.1002/ece3.5245)
Supplement: Supplementary file 1 [file ECE3-9-6655-s001.docx]

Supplementary data

Table S1. Summary of number of sampling points, orchid taxa and number of specimens found in cemeteries and roadside verges in Crete, Cyprus and Lesbos during both thematic and non-thematic sampling.

|  | Cyprus | | | | Crete | | | | Lesbos | | | |  |
| --- | --- | --- | --- | --- | --- | --- | --- | --- | --- | --- | --- | --- | --- |
|  | Cemeteries | | Roadsides | | Cemeteries | | Roadsides | | Cemeteries | | Roadsides | | Total number of individuals |
| Species | No. of cemeteries | Total no. of individuals | No. of roadsides | Total no. of individuals | No. of cemeteries | Total no. of individuals | No. of roadsides | Total no. of individuals | No. of cemeteries | Total no. of individuals | No. of roadsides | Total no. of individuals |  |
| *Anacamptis pyramidalis* (L.) L.C.M. Richard | 1 | 62 |  |  | 1 | 10 | 13 | 117 |  |  | 2 | 10 | 199 |
| *Cephalanthera longifolia* (L.) Fritsch |  |  |  |  |  |  |  |  |  |  | 1 | 2 | 2 |
| *Dactylorhiza romana* (Sebastiani) Soó |  |  | 3 | 6 |  |  |  |  |  |  |  |  | 6 |
| *Epipactis veratrifolia*Boissier and Hohenacker |  |  | 1 | 11 |  |  |  |  |  |  |  |  | 11 |
| *Himantoglossum comperianum*(Steven) P. Delforge |  |  |  |  |  |  |  |  |  |  | 1 | 4 | 4 |
| *Himantoglossum caprinum* (M.-Bieb) Sprengel |  |  |  |  |  |  |  |  |  |  | 1 | 2 | 2 |
| *Himantoglossum robertianum*(Loiseleur) P. Delforge | 7 | 24 | 56 | 307 |  |  | 26 | 230 |  |  |  |  | 561 |
| *Limodorum abortivum* (L.) Swartz |  |  | 1 | 6 |  |  |  |  |  |  | 2 | 4 | 10 |
| *Ophrys alasiatica*Kreutz, Segers and H. Walraven |  |  | 24 | 230 |  |  |  |  |  |  |  |  | 230 |
| *Ophrys ariadnae*H.F. Palus |  |  |  |  |  |  | 5 | 10 |  |  |  |  | 10 |
| *Ophrys attica* (Boissier and Orphanides) B.D. Jackson |  |  | 8 | 46 |  |  |  |  |  |  |  |  | 46 |
| *Ophrys bombyliflora* Link |  |  |  |  |  |  | 6 | 62 |  |  |  |  | 62 |
| *Ophrys bornmuelleri* M. Schulze |  |  | 2 | 2 |  |  |  |  |  |  |  |  | 2 |
| *Ophrys ceto* P. Devillers, J. Devillers-Tercshuren and P. Delforge |  |  |  |  |  |  |  |  |  |  | 1 | 1 | 1 |
| *Ophrys bucephala* Gölz andH. R. Reinhard |  |  |  |  |  |  |  |  |  |  | 1 | 2 | 2 |
| *Ophrys cinereophila* H.F. Paulus and Gack | 1 | 1 | 5 | 13 | 1 | 1 | 5 | 32 |  |  |  |  | 47 |
| *Ophrys creberrima* H.F. Paulus |  |  |  |  |  |  | 1 | 38 |  |  |  |  | 38 |
| *Ophrys cretensis* (H.Baumann and Künkele) H.F. Paulus |  |  |  |  |  |  | 11 | 71 |  |  |  |  | 71 |
| *Ophrys creticola* H.F. Paulus |  |  |  |  |  |  | 1 | 1 |  |  |  |  | 1 |
| *Ophrys cretica* (Vierhapper) E. Nelson |  |  |  |  |  |  | 19 | 72 |  |  |  |  | 72 |
| *Ophrys elegans* (Renz) H. Baumann and Künkele |  |  | 2 | 4 |  |  |  |  |  |  |  |  | 4 |
| *Ophrys episcopalis* Poiret |  |  |  |  | 1 | 1 | 3 | 8 |  |  |  |  | 9 |
| *Ophrys ferrum-equinuum* Desfontaines |  |  |  |  |  |  |  |  |  |  | 1 | 1 | 1 |
| *Ophrys flavomarginata* (Renz) H. Baumann and Künkele | 1 | 150 | 20 | 109 |  |  |  |  |  |  |  |  | 259 |
| *Ophrys gortynia* (H. Baumann and Künkele) H.F. Paulus |  |  |  |  | 2 | 3 | 11 | 28 |  |  |  |  | 31 |
| *Ophrys heldreichii* Schlechter |  |  |  |  |  |  | 9 | 69 |  |  |  |  | 69 |
| *Ophrys herae* Hirth and Spaeth |  |  | 2 | 2 |  |  | 1 | 1 |  |  |  |  | 3 |
| *Ophrys helenae* Renz |  |  |  |  |  |  |  |  |  |  | 1 | 1 | 1 |
| *Ophrys homeri* Hirth and Spaeth |  |  |  |  |  |  |  |  |  |  | 5 | 41 | 41 |
| *Ophrys iricolor* Desfontaines |  |  | 6 | 37 |  |  | 3 | 54 |  |  |  |  | 91 |
| *Ophrys israelitica* H. Baumann and Künkele |  |  | 7 | 66 |  |  |  |  |  |  |  |  | 66 |
| *Ophrys kotschyi* H. Fleischmann and Soó |  |  | 1 | 2 |  |  |  |  |  |  |  |  | 2 |
| *Ophrys lapethica* Gölz and H.R. Reinhard |  |  | 3 | 4 |  |  |  |  |  |  |  |  | 4 |
| *Ophrys lesbis* Gölz and H.R. Reinhard |  |  |  |  |  |  |  |  |  |  | 1 | 1 | 1 |
| *Ophrys levantina* Gölz and H.R. Reinhard |  |  | 7 | 70 |  |  |  |  |  |  |  |  | 70 |
| *Ophrys lutea* Cavanilles |  |  |  |  |  |  | 1 | 1 |  |  |  |  | 1 |
| *Ophrys mammosa* Desfontaines | 1 | 42 | 4 | 16 |  |  | 1 | 19 |  |  | 5 | 13 | 90 |
| *Ophrys morio* H.F. Paulus and Kreutz |  |  | 1 | 1 |  |  |  |  |  |  |  |  | 1 |
| *Ophrys minutula* Gölz and H.R. Reinhard |  |  |  |  |  |  |  |  |  |  | 2 | 4 | 4 |
| *Ophrys oestrifera* Steven in M.-bieb |  |  |  |  |  |  |  |  |  |  | 1 | 1 | 1 |
| *Ophrys omegaifera* H. Fleischmann |  |  |  |  |  |  | 6 | 44 |  |  | 2 | 2 | 46 |
| *Ophrys phryganea* J. Devillers-Terschuren and P. Devillers |  |  |  |  |  |  | 3 | 13 |  |  |  |  | 13 |
| *Ophrys sicula* Tineo |  |  | 9 | 36 | 3 | 37 | 21 | 120 |  |  | 15 | 148 | 341 |
| *Ophrys sitiaca* H. Paulus, Ch. Alibertis and A. Alibertis |  |  |  |  |  |  | 1 | 7 |  |  |  |  | 7 |
| *Ophrys speculum* Link |  |  |  |  |  |  |  |  |  |  | 1 | 1 | 1 |
| *Ophrys spruneri* Nyman |  |  |  |  |  |  | 2 | 5 |  |  |  |  | 5 |
| *Ophrys tenthredinifera* Willdenow |  |  |  |  |  |  | 1 | 7 |  |  |  |  | 7 |
| *Ophrys umbilicata* Desfontaines |  |  | 13 | 32 |  |  |  |  |  |  |  |  | 32 |
| *Ophrys villosa* Desfontaines |  |  |  |  |  |  | 6 | 47 |  |  |  |  | 47 |
| *Orchis anatolica* Boissier |  |  | 1 | 16 |  |  | 1 | 5 |  |  |  |  | 21 |
| *Orchis anthropophora* (L.) Allioni |  |  |  |  |  |  | 2 | 6 |  |  |  |  | 6 |
| *Orchis boryi* Reichenbach fil. |  |  |  |  |  |  | 1 | 4 |  |  |  |  | 4 |
| *Orchis collina*Banks and Solander ex Russel | 3 | 102 | 13 | 55 | 1 | 3 | 14 | 254 |  |  | 1 | 9 | 423 |
| *Orchis fragrans*Pollini |  |  | 11 | 101 | 1 | 500 | 7 | 176 |  |  |  |  | 777 |
| *Orchis intacta* Link | 3 | 47 | 12 | 52 |  |  |  |  |  |  | 2 | 4 | 103 |
| *Orchis italica* Poiret | 1 | 2 | 16 | 75 | 3 | 9 | 23 | 82 |  |  | 3 | 8 | 176 |
| *Orchis lactea* Poiret |  |  |  |  |  |  | 5 | 58 |  |  |  |  | 58 |
| *Orchis laxiflora* Lamarck |  |  |  |  |  |  | 10 | 67 |  |  | 1 | 6 | 73 |
| *Orchis papilionacea* L. |  |  |  |  | 3 | 28 | 19 | 62 |  |  |  |  | 90 |
| *Orchis pauciflora* Tenore |  |  |  |  |  |  | 1 | 2 |  |  |  |  | 2 |
| *Orchis picta* Loiseleur |  |  |  |  |  |  |  |  |  |  | 4 | 22 | 22 |
| *Orchis punctulata* Steven ex Lindley |  |  | 2 | 3 |  |  |  |  |  |  |  |  | 3 |
| *Orchis purpurea* Hudson |  |  |  |  |  |  |  |  |  |  | 1 | 1 | 1 |
| *Orchis quadripunctata* Cyrillo ex Tenore |  |  |  |  | 1 | 20 | 8 | 32 |  |  |  |  | 52 |
| *Orchis sancta* L. |  |  | 11 | 87 |  |  |  |  | 3 | 30 | 62 | 578 | 695 |
| *Orchis simia* Lamarck |  |  |  |  |  |  | 5 | 85 |  |  | 1 | 11 | 96 |
| *Orchis syriaca* Boissier ex. H. Baumann and Künkele | 1 | 2 | 12 | 46 |  |  |  |  |  |  |  |  | 48 |
| *Orchis tridentata* Scopoli |  |  |  |  |  |  |  |  |  |  | 3 | 7 | 7 |
| *Orchis troodi* (Renz) P. Delforge |  |  | 13 | 1 |  |  |  |  |  |  |  |  | 1 |
| *Platanthera holmboei* H. Lindberg fil |  |  | 1 | 4 |  |  |  |  |  |  |  |  | 4 |
| *Serapias bergoniii* E.G. Camus | 1 | 1 | 26 | 276 | 2 | 215 | 42 | 354 |  |  |  |  | 846 |
| *Serapias cordigera* L. |  |  |  |  | 1 | 2 | 10 | 142 |  |  | 1 | 9 | 153 |
| *Serapias lingua* L. |  |  |  |  |  |  | 10 | 82 |  |  |  |  | 82 |
| *Serapias orientalis* (Greuter) H. Baumann and Künkele |  |  |  |  | 2 | 5 | 5 | 15 |  |  | 2 | 5 | 25 |
| *Serapias parviflora* Parlatore |  |  |  |  |  |  | 84 | 11 |  |  | 34 | 184 | 195 |
| *Serapias politisii* Renz |  |  |  |  |  |  |  |  | 2 | 2 |  |  | 2 |
| *Spiranthes spiralis* (L.) Chevallier |  |  |  |  |  |  | 1 | 1 | 1 | 3 | 4 | 9 | 13 |
| Total | 20 | 433 | 293 | 1716 | 22 | 834 | 152 | 2494 | 6 | 35 | 162 | 1091 |  |
